# New Perspectives on Circulating Ferritin: Its Role in Health and Disease

**DOI:** 10.3390/molecules28237707

**Published:** 2023-11-22

**Authors:** Óscar Fonseca, Ana S. Ramos, Leonor T. S. Gomes, Maria Salomé Gomes, Ana C. Moreira

**Affiliations:** 1 i3S—Instituto de Investigação e Inovação em Saúde, Universidade do Porto, 4200-135 Porto, Portugal; ofonseca@i3s.up.pt (Ó.F.); aramos@i3s.up.pt (A.S.R.); leonorg@i3s.up.pt (L.T.S.G.); sgomes@i3s.up.pt (M.S.G.); 2 FCUP—Faculdade de Ciências, Universidade do Porto, 4169-007 Porto, Portugal; 3 ICBAS—Instituto de Ciências Biomédicas Abel Salazar, Universidade do Porto, 4050-313 Porto, Portugal; 4 IBMC—Instituto de Biologia Molecular e Celular, Universidade do Porto, 4200-135 Porto, Portugal

**Keywords:** iron metabolism, ferritin, disease, biomarker, therapy

## Abstract

The diagnosis of iron disturbances usually includes the evaluation of serum parameters. Serum iron is assumed to be entirely bound to transferrin, and transferrin saturation—the ratio between the serum iron concentration and serum transferrin—usually reflects iron availability. Additionally, serum ferritin is commonly used as a surrogate of tissue iron levels. Low serum ferritin values are interpreted as a sign of iron deficiency, and high values are the main indicator of pathological iron overload. However, in situations of inflammation, serum ferritin levels may be very high, independently of tissue iron levels. This presents a particularly puzzling challenge for the clinician evaluating the overall iron status of the patient in the presence of an inflammatory condition. The increase in serum ferritin during inflammation is one of the enigmas regarding iron metabolism. Neither the origin, the mechanism of release, nor the effects of serum ferritin are known. The use of serum ferritin as a biomarker of disease has been rising, and it has become increasingly diverse, but whether or not it contributes to controlling the disease or host pathology, and how it would do it, are important, open questions. These will be discussed here, where we spotlight circulating ferritin and revise the recent clinical and preclinical data regarding its role in health and disease.

## 1. Introduction

Iron is one of the most abundant elements on Earth. Its importance to life is evident from the fact that it is essential for most known living organisms, to the extent that even *Lactobacillaceae*, once considered to be iron-independent microorganisms, are now known to require this element for growth under certain circumstances [1]. Iron is a key element in a wide variety of biological processes, acting as a co-factor for proteins responsible for cell proliferation and differentiation, mitochondrial respiration, nucleic acids synthesis, and oxygen transport, among others [2]. There are several diseases associated with alterations in the iron metabolism, which encompass a broad spectrum of clinical manifestations. A lack of iron in circulation is typically associated with the iron deficiency anemia disease, which mostly affects preschool children and women in their reproductive age [3]. On the other hand, the excess of iron in circulation leads to tissue iron overload disorders. Due to its redox activity, excessive iron accumulation induces cytotoxic effects, as iron also catalyzes the production of reactive oxygen species (ROS) and lipid peroxidation. This production has deleterious consequences for the cells, as observed in ferroptosis events [4]. Although alterations in iron homeostasis can be indirectly detected by the evaluation of serum iron parameters, the assessment of tissue iron status can be also performed using magnetic resonance imaging [5].

Acting as an indispensable nutrient, iron is also required by macro- and microorganisms. Thus, it is comprehensible that a quest for iron arises when both organisms interact, as this occurs in human infections caused by bacteria in which the pathogen aims to acquire iron from the host, establish the infection, and secure its proliferation and survival [6]. The pathogens use specific iron-chelating biomolecules termed siderophores to scavenge iron from their surroundings [7]. To counterbalance this, the host produces siderophore-binding proteins, such as lipocalin 2, thereby limiting iron accessibility to pathogens [8]. Furthermore, the existence of mammalian siderophores involved in iron transport has also been proposed [9].

While dietary iron is mainly absorbed by the enterocytes, iron recycling is mostly managed by the macrophages. Both types of cells release iron into circulation, which will be further stored in the hepatocytes, the major site for iron deposit [10]. The processes of iron import, export, storage, and delivery are coordinated by a highly regulated protein network. Serum iron is mostly bound to transferrin (Tf), which serves as a major vehicle for iron delivery into cells. Transferrin saturation (Tfsat) levels have been widely used to characterize iron metabolism disorders, because low or high Tf saturation levels indicate iron deficiency or iron overload conditions, respectively [11,12].

Iron ions at physiological pH (7.4) exist mostly in two different oxidation states—ferrous (Fe^2+^) and ferric (Fe^3+^) iron—and their bioavailability is influenced by their hydrolytic profile, reflecting the tendency to form iron hydroxide complexes [13]. Thus, changes in the pH surrounding iron can affect its solubility and bioavailability. Although mammalians are equipped with compensatory mechanisms that balance changes in the pH, disease conditions, such as chronic kidney disease [14] and sepsis [15], may compromise this regulation.

Clinical evaluation of the iron status of an individual largely relies on the measurement of serum parameters [16], including iron, transferrin, soluble transferrin receptor, and Tfsat [6]. Routine clinical laboratory determinations may not include the quantification of Tf. Instead, iron itself is quantified after being released from serum proteins by pH manipulation, and the total iron-binding capacity of serum is evaluated by determining the total amount of iron that can be bound to all of the proteins present [16]. In this way, the nature of the serum iron-binding proteins may not be directly determined. Another important parameter measured as part of the evaluation of iron status is serum ferritin (sFT). Although it is mainly known as an intracellular iron storage protein, FT can also be found in serum [17]. In the absence of inflammatory conditions, the amount of sFT is considered a surrogate of the overall tissue iron levels. Low sFT values are interpreted as a sign of iron deficiency, and high sFT values are the main indicator of pathological iron overload. In situations of inflammation, however, sFT levels may be very high, irrespective of tissue iron levels [18].

We previously reviewed the role of ferritin as an inflammatory player in host–pathogen interaction [19]. Here, we assess the current knowledge regarding circulating FT and its possible role in health and disease, focusing on publications from the last 3 years. Thus, we start with a contextualization of systemic iron and iron homeostasis (Section 2), then we discuss ferritin structure and regulation (Section 3). Subsequently, we go on to highlight circulating ferritin and the recent advancement in this area of study (Section 4), and we end this review by considering the possibility of using ferritin as a therapeutic target (Section 5).

## 2. Circulating Iron

**Figure 1:**
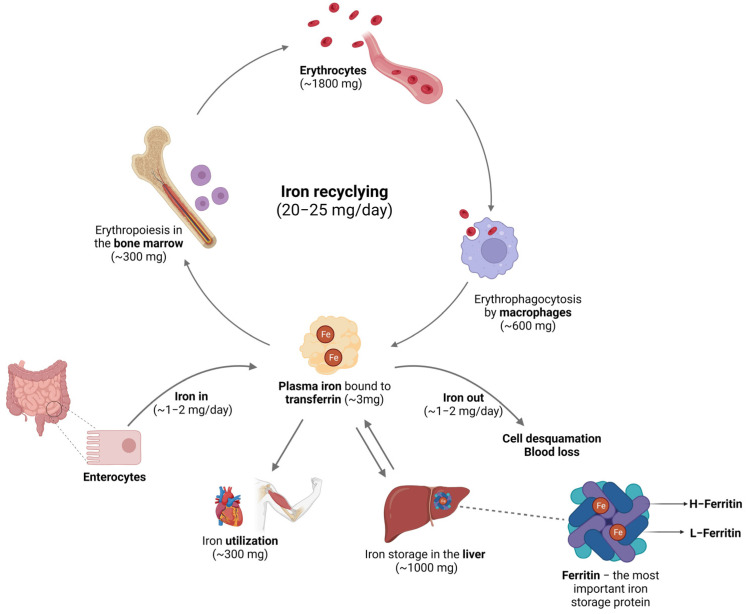
Iron in circulation. In mammalians, iron from the diet is absorbed through enterocytes, and it can be lost through cell desquamation and blood loss. Circulating iron is bound to Tf, used in the erythropoiesis process, and recycled by the macrophages after phagocytosis of old or senescent red blood cells. For storage purposes, iron is kept mostly in the hepatocytes inside the FT nanostructure. Image created with BioRender (www.biorender.com, accessed on 15 September 2023).

Maintaining balanced circulating levels of iron is crucial for the overall homeostasis of organisms, as both deficient and excessive amounts of iron can disrupt normal physiological processes. To ensure proper iron regulation and prevent the development of pathological conditions, intricate mechanisms are in place to tightly control iron storage, absorption, and distribution. These processes are interrelated, as depicted in Figure 1, and they are governed by a network of proteins that operates at both systemic and cellular levels in vertebrates [19], as discussed below.

**Figure 2:**
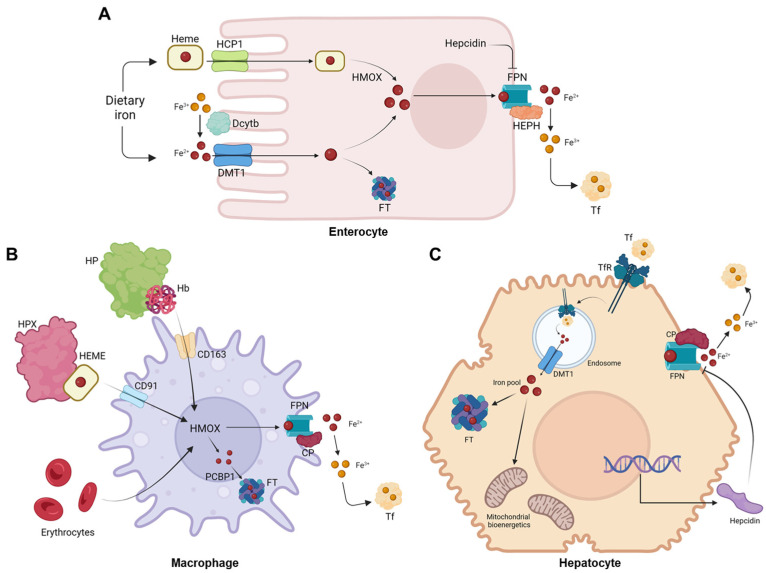
Cellular iron metabolism. (**A**) Dietary iron is absorbed by the enterocytes. (**B**) Macrophages participate in the process of iron recycling from the erythrocytes. (**C**) The hepatocytes act as the major cell site for iron storage. Abbreviations: CD163—Cluster of differentiation 163, hemoglobin-haptoglobin receptor; CD91—Cluster of differentiation 91, also known as Low-Density Lipoprotein Receptor-Related Protein 1 (LRP1) or α2-macroglobulin receptor; CP—Ceruloplasmin; Dcytb—Duodenal cytochrome B; DMT1—Divalent metal transporter 1; FPN—Ferroportin; FT—Ferritin; Hb—Hemoglobin; HCP1—Heme carrier protein 1; HEPH—Hephaestin; HMOX—Heme oxygenase; HP—Haptoglobin; HPX—Hemopexin; PCBP1—Poly(RC) binding protein 1; Tf—Transferrin; TfR—Transferrin receptor. Image created with BioRender (www.biorender.com, accessed on 15 September 2023).

The body can maintain relatively constant iron levels due to the constant recycling of the iron present in red blood cells. Mammals lack active iron excretion systems, and only minor amounts are lost through certain processes, such as skin desquamation, sloughing of intestinal epithelial cells, bleeding, and through urine [6]. To compensate for these small losses, individuals obtain iron from their diet—typically, around 1–2 mg per day. The absorption of iron primarily occurs in the enterocytes (Figure 2A), and it is tightly regulated by various factors [20]. Non-heme iron is transported across the apical membrane of the intestinal cells via DMT1. However, because this transporter only internalizes metals in their 2^+^ reduction state, dietary iron, which is usually in the 3^+^ form (Fe^3+^), must first be reduced by a ferrireductase known as duodenal cytochrome b (Dcytb) [21]. However, the mechanisms responsible for the absorption of iron in its heme form are yet to be determined. Once inside the enterocytes, heme is broken down by heme oxygenase 1 (Hmox-1), resulting in the release of free iron, carbon monoxide, and bilirubin [6]. In its free form, iron undergoes similar processes to inorganic dietary iron [2]. Following its absorption by enterocytes, iron can essentially have different destinations. It can be either transported or stored, depending on the organism’s requirements for this mineral. Most of the iron is used for erythropoiesis within the bone marrow cavity and enters circulation contained in erythrocytes’ heme complexes. When these cells are damaged or become senescent, they are phagocytosed by macrophages (Figure 2B), which will recover the heme iron and ensure the recycling processes that will make iron available for the formation of new erythrocytes or other bodily needs [22]. Regarding iron storage, hepatocytes (Figure 2C) are the key cells ensuring that iron is either properly safekept inside the FT nanocages or exported into circulation to meet metabolic requirements [10]. The export of iron from these cells is facilitated by ferroportin (FPN), a transmembrane protein that serves as the sole known iron exporter in mammalian cells [23]. The cell surface levels of FPN are regulated by hepcidin, a peptide hormone produced by the liver that plays a critical role in iron homeostasis. Initially described as an antimicrobial peptide [24,25], hepcidin primarily regulates the flux of iron in tissues by promoting the degradation of FPN [26].

In conditions of iron overload, hepcidin levels increase, leading to a decrease in FPN levels [27]. Consequently, iron export is reduced, resulting in increased intracellular iron storage and reduced circulating iron levels. Conversely, in anemic or hypoxic conditions, hepcidin levels decrease [28]. This leads to reduced degradation and higher cell surface expression of FPN, allowing for greater iron efflux to elevate circulating iron levels. Iron released by FPN undergoes oxidation through the action of the ferroxidase ceruloplasmin, converting Fe^2+^ to Fe^3+^ once again. The resulting Fe^3+^ is then bound to Tf [29]. In humans, Tf is a single polypeptide chain of 76 kDa predominantly synthesized in the liver. It comprises two globular domains at the N- and C-termini, each capable of binding one iron ion with high affinity. This binding is reversible, enabling each molecule of Tf to carry two Fe^3+^ ions in the bloodstream [30].

Under basal conditions, cells internalize iron from transferrin through transferrin receptors (TfR). Transferrin–iron complexes bind to TfR with high affinity and are internalized through endocytosis. Within the acidic environment of the endosome, Tf releases iron, which is subsequently exported to the cytosol through DMT1, expressed in the endosomal membrane, following reduction. Holo-Tf then returns to the bloodstream [31]. While this is the primary mechanism by which cells acquire iron, TfR-independent iron uptake has also been reported in in vitro studies [32,33,34].

## 3. Ferritin

**Figure 3:**
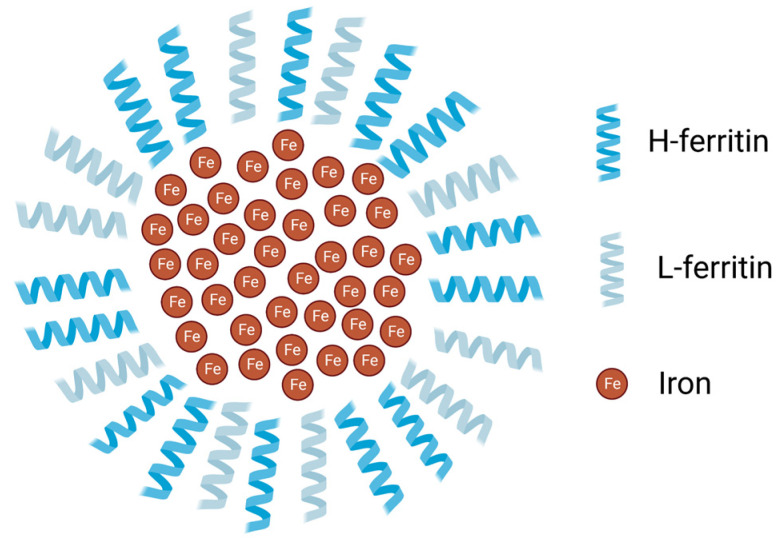
Scheme of ferritin’s heteropolymer. The 24-meric shell is constituted by L- and H-ferritin subunits formed by 4 alpha-helices and a short helix on top (for simplicity, represented here by one helix each) and lodges iron in its core. The ratio of each peptide varies depending on the cell type. Image created with BioRender (www.biorender.com, accessed on 15 September 2023).

Ferritin is a central protein in iron metabolism, as previously mentioned, as it stores iron inside the cells and protects them from the damaging properties of this element [35]. Regarding its structure, FT is composed of two different subunits—L- and H-ferritin (Figure 3)—and each subunit is composed of four α-helices that constitute a bundle and a fifth helix laying at an acute angle to the bundle [36]. L-ferritin (FTL) has a molecular weight of 19 kDa and plays a structural role, helping in mineralization as well. H-ferritin (FTH) has a molecular weight of 21 kDa and has ferroxidase activity, being able to bind Fe^2+^ in a dinuclear binding site [37]. The reaction catalyzed by FTH allows for the conversion of Fe^2+^ into Fe^3+^, the iron form that can be incorporated by this storage protein [38]. FT is composed of 24 subunits that form a spheric complex with an inner diameter of 8 nm and an outer diameter of 12 nm [39], storing iron within the nanocage structure [40]. When needed, iron is released from ferritin by still-unknown mechanisms, which may include ferritin degradation in the lysosomes. The proportions of L and H peptides may vary according to the cell type, physiological state, and response to infection/inflammation [13].

The two subunits co-assemble in various proportions to form a 24-meric hollow cage. The protein can accommodate a large number of iron atoms in its core, with most experimental data indicating a limit of around 3000 atoms [41,42]. Iron can enter the nanocage through six hydrophobic four-fold channels, as well as eight hydrophilic three-fold channels that can be found throughout the protein [42].

As previously mentioned, FT is mainly an intracellular protein, and it is mostly found in the cytosol and mitochondria [43]. Importantly, it is known that FT may be present in the serum. Given the absence of a secretory peptide on the protein’s sequence, it is thought that it leaves the cells through an ER- and Golgi-independent non-classical secretory pathway [44,45].

Furthermore, it has already been described that FTH is not only essential for embryonic development [46], but also that myeloid cells lacking the gene coding for this protein are more susceptible to iron-induced oxidative stress [47]. As we previously reviewed, FT has a role in different contexts of disease, such as cancer, neurodegenerative diseases, and infections, among others [19]. FT is an acute-phase protein, and its serum levels have been used not only as an iron status marker but also as a biomarker of inflammatory conditions [48]. In fact, sFT levels have been associated with the severity of COVID-19 disease progression, correlating higher FT levels with fatal outcomes over time [49]. Other studies have pointed out that circulating FT levels are associated with dysfunctions of the adipose tissue [50,51] and linked with increased risk of cardiovascular disturbances [52]. Moreover, because FT secretion is stimulated by pro-inflammatory cytokines and FTH has pro-inflammatory effects, it has been hypothesized that elevated FT levels in the serum may not only be a consequence of inflammation but also a part of the pathogenic mechanism of the disease, thus contributing to the observed inflammatory burden [53,54].

### Cell-Intrinsic Regulation and Modulation

The most prominent form of regulation of ferritin expression at the cellular level is the IRE-IRP system. It is composed of the iron regulatory proteins (IRPs), which respond to cellular iron levels, and the iron responsive elements (IREs) [13]. The IREs are hairpin structures localized in the untranslated regions of mRNA encoding for proteins related to iron metabolism, including FT. IREs can be localized either in the 5′ UTR, impairing the translation of the mRNA, or in the 3′ UTR, stabilizing the nucleic acid and, consequently, allowing it to be translated more extensively [13]. In the case of iron depletion in the cell, the IRPs bind to IREs. As the IREs of the mRNA that codify FT are localized in the 5′ UTR, there is a downregulation of these proteins, thus allowing iron to be free for usage [55].

Besides the downregulation of FT translation, when there is a shortage of iron in the cell, FT releases the mineral, making it available for consumption. For this to happen, the storage protein must be transported into autophagosomes by cargo nuclear receptor coactivator 4 (NCOA4). There, the protein will undergo a process called ferritinophagy, thereby releasing iron [56].

Intracellular iron levels are not the only factor that influences the expression of FT. This process may also be regulated during inflammation and infection. More precisely, it has been shown that several cytokines, including Interleukin-1β, IL-6 [57,58], and tumor necrosis factor-alpha [59,60], induce the expression of both *Ftl* and *Fth* by promoting the activation of the nuclear factor-kB. Furthermore, upon in vitro infection with *Mycobacterium avium*, *Fth* transcription is induced in macrophages by a process mediated by the Toll-like receptor 2 [61]. Inflammation and the IRE-IRP system are interconnected, as observed, for example, in the role of interferon-gamma and lipopolysaccharide, which induce the degradation of IRP2 and lead to an increase in FT synthesis in macrophages [62]. In contrast, IL-10, an anti-inflammatory cytokine, may also upregulate FT’s expression, as it was shown in patients with inflammatory bowel disease [63], hemophagocytic lymphohistiocytosis [64], or rapidly progressive interstitial lung disease [65].

## 4. Circulating Ferritin

Serum ferritin levels can be used as an indicator of tissue iron levels. It is also considered an acute-phase protein. Hyperferritinemia, which refers to elevated sFT levels, is recognized as one of the serum markers of inflammation, although its precise contribution to pathology or infection resolution is still under investigation [19,66]. While it may not be an efficient iron carrier protein compared to Tf, it can carry larger amounts of iron, highlighting its potential role as an iron delivery system that should not be disregarded [44].

The role of serum FT and its subunits ratio, iron content, and pathways of cellular export are not fully understood. Some studies claim that serum FT is formed mainly by FTL subunits with a few FTH subunits and, thus, it contains a small amount of iron due to the poor ferroxidase activity. In contrast, other studies argue that sFT presents significant iron content [44]. Moreover, regarding pathways of cellular export, there are two main views: some authors have shown that intracellular FT is passively released during the death of damaged cells [67], and others have shown data that support that it is actively released by some cells, such as macrophages, through non-classical secretory autophagy and multivesicular body–exosome pathways. It is noteworthy that it has been proposed that human sFT has mannose- or glucose-like molecules on the protein surface, which may be a marker for endoplasmic reticulum–Golgi processing [44,68]. We have recently shown that FTH produced by myeloid cells plays a crucial role in iron redistribution during mycobacterial infection, as it is essential for iron deposition and storage inside macrophages. In the absence of myeloid-derived FTH, a high FPN expression in macrophages leads to iron export from these cells and accumulation in the tissue parenchyma (namely, in hepatocytes) [66]. However, how FT is released into circulation during infection is not yet known. Recently, it was suggested that extracellular vesicles (EVs) may play a role in this context. EVs are small nanoparticles released by various cell types in both physiological and disease conditions. Their main function is to support local and long-distance cell–cell communication [69]. It has been described that upon iron loading, fibroblasts secrete iron-containing FT, both H and L subunits, through CD63-positive EVs [70]. Moreover, the idea that FT is secreted via the exosome pathway has been corroborated by additional data [68]. It is of note that an augmented secretion of CD63 + EVs has also been demonstrated in the case of infection with herpes simplex virus 1 [71]. On top of that, a secretion of iron-loaded FT by EVs in in vivo models has also been reported. For example, it was shown that sow’s milk contains EVs loaded with iron-bound FT [72] and that mice’s and *Drosophila melanogaster’s* oligodendrocytes secrete EVs containing FTH and Ferritin 1 heavy chain homologue (Fer1HCH), respectively [73]. Therefore, the role of EVs in the release of ferritin to circulation should be further investigated.

### Role of Circulating Ferritin in Health and Disease

It is now clear that serum ferritin is an acute-phase protein. It is thus expected that it can become a useful biomarker. But, this protein’s origin, and the role it plays, are not yet totally disclosed. Even though high or low sFT levels during inflammatory conditions may not correlate with the tissue iron content [18], sFT is still being used to evaluate the state and progression of some infectious and/or inflammatory pathologies, as it acts as a biomarker or as a prognostic indicator in several diseases. sFT’s importance in health and disease may also be focused on the intracellular signaling pathways in which the protein is involved. Intracellular FT has been linked to the development of ferroptosis in several models of disease, such as chronic obstructive pulmonary disease [74] or heart failure driven by cardiomyopathies [75,76]. It has been described that ferroptosis might be driven by autophagy processes, in which cells destroy their dysfunctional components. However, autophagy has also been understood as a type of cell death mechanism (reviewed in [77,78]), and it has been related to FT because the destruction of the protein in the process of ferritinophagy leads to the increase of labile iron and the consequent production of ROS, which will ultimately lead to oxidative cell death [4].

In this context, the pathologies associated with the cardiovascular system have been defined by the World Health Organization as the leading cause of mortality worldwide, making them essential to be discussed [79]. Despite the current knowledge regarding the cardiovascular risk posed by increased amounts of iron in circulation [80], a direct and robust correlation between high sFT levels and increased cardiovascular risk is still missing. Nonetheless, a very recent study has indicated that there was no association between sFT levels and the risk of developing cardiovascular disease or congestive heart failure [81]. Also, elevated sFT levels may be linked to a higher risk of cerebrovascular disease [82], even though more research in the field is required. Patients undergoing peritoneal dialysis showed an association between cardiovascular mortality and higher sFT [83]. Moreover, a study has pointed out that in patients at risk of obesity, high sFT is associated with the prevalence of cardiovascular disease risk factors [84]. Interestingly, an example of a disease with cardiovascular involvement in which sFT levels are used as a biomarker is Kawasaki disease. Patients with this condition experience acute febrile systemic vasculitis, in which their blood vessels become highly inflamed. Although it was already reported that sFT levels are elevated during Kawasaki disease [85], it was also recently shown that sFT may be used in the differential diagnosis between Kawasaki disease and other febrile pathologies, and also in the prognosis of coronary artery lesions in patients with this condition [86,87].

In patients with transfusion-dependent thalassemia, serum ferritin levels correlate not only with tissue iron overload but also with immune dysregulation. A significant correlation was found between sFT and the frequency of T regulatory cells and myeloid-derived suppressor cells [88].

FT has been proven to be a reliable biomarker in cancer patients due to its elevated serum levels when compared to healthy individuals. Lately, several studies have correlated the increased amounts of this protein with the higher risk of developing oncologic disorders, as well as prognosing the positive or negative advance of the disease. Regarding breast cancer, increased sFT has been positively associated with the increased incidence of the disease [89], and even with the severity of cancer progression [90]. Increased levels of FT are also a good indication of an escalated risk of pancreatic cancer development [91]. Moreover, sFT has also been associated with sarcopenia in patients with gastric cancer [92]. Serum ferritin concentration has been described as a reliable prognostic indicator in other oncologic pathologies, such as multiple myeloma [93], lung cancer [94], and Hodgkin lymphoma [95].

As previously mentioned, iron is an important nutrient for all living organisms, and, among its variety of functions, its role in the brain tissue must not be disregarded. The accumulation of iron in the brain tissue has been correlated with the development of neurodegeneration processes, mostly due to the occurrence of Fenton’s reactions and neuronal cell death caused by oxidative stress [96]. It has been described that FTH has a protective role against ferroptosis events in the neuronal tissue of a mouse model of traumatic brain injury [97] and in a rat model of Parkinson’s disease [98]. In Alzheimer’s disease, for example, FT has been pointed out to be a potential biomarker for the understanding of microglia activity, because its circulating cerebrospinal fluid levels are significantly correlated with the increased levels of sTrem2, a marker of neuroinflammation and neurodegeneration [99].

Hyperferritinemia is associated with various metabolic and inflammatory disorders. sFT has been characterized as a possible biomarker for predicting the development of non-alcoholic fatty liver disease [100,101], type 2 diabetes mellitus [102], and obesity [103], and also for envisaging the progression of myalgic encephalomyelitis/chronic fatigue syndrome in COVID-19 patients [104]. Moreover, sFT levels were associated with the development of liver fibrosis in patients with autoimmune hepatitis [105], although these correlations were contradicted by another study published later in the same year [106]. Furthermore, and regarding its role as a predictive biomarker, sFT has been implicated in the development of COVID-19 infections [49,107], correlating its serum levels with a more severe and deadly profile of the disease [108,109]. It has been reported that repeated measurements of sFT levels in COVID-19 patients were useful for predicting the trajectory of the disease, allowing a better allocation of the intensive care resources based on personalized medicine [110]. However, to use FT levels as a reliable predictive biomarker, a careful search for other co-morbidities must be addressed in order to understand if the inflammatory trigger that raises FT levels is caused by the viral infection or not. Recently, a direct molecular relationship between hyperferritinemia and Adult-onset Still disease was discovered. In fact, in the context of this disease, it was shown that ferritin can act on Msr1 at the surface of neutrophils, triggering an excessive neutrophil leukocyte infiltration and neutrophil extracellular trap formation [111]. Although this was described in Adult-onset Still disease, it may open the door to a better understanding of not only the molecular mechanisms linking hyperferritinemia and inflammatory diseases, but the design of therapeutic approaches targeting these pathways. Interestingly, aceruloplasminemia, which is a rare, adult-onset, autosomal recessive disorder characterized by systemic iron overload due to mutations in the Ceruloplasmin gene, is usually associated with mild anemia and with hyperferritinemia. This increase in ferritin usually occurs before the beginning of symptoms, and it is essential to prevent the severe progression of the disease (reviewed in [112]).

## 5. Circulating Ferritin as a New Therapeutic Strategy

Although there are no well-established approaches involving ferritin to treat disease yet, there is evidence that ferritin and its mechanisms of action may play a role in therapeutic strategies. During infection, pathogens try to obtain iron from the host for their proliferation and survival, leading to alterations in the host’s iron metabolism. Infectious diseases remain a persistent health problem worldwide, despite the reduction of the burden of disease when antimicrobial treatments were introduced [113]. The current prevalence of infectious diseases is influenced by the evolution of microbe strains resistant to drug therapies [114]. Thus, new therapeutical approaches are required to treat these infections.

The use of host-directed therapies arises as a novel approach [115]. These therapeutic strategies aim to target host players involved in the pathogenic pathways of the disease, thus altering its course and avoiding the antimicrobial resistance problematic. Furthermore, host-directed therapy may also improve the defense mechanisms while simultaneously reducing the effects of exacerbated inflammation [116,117]. Moreover, the combination of host-directed therapies with pathogen-directed treatments, like antibiotics or antimicrobial peptides, makes it possible to synergize the beneficial effects of both therapeutics, thereby decreasing the drug dose administrated, minimizing its toxicity, and avoiding antimicrobial drug resistance [118,119,120].

The blocking of iron acquisition has been proven to be a promising therapeutic strategy against bacterial infections, such as the ones caused by mycobacteria. Recently, a new clinical approach reducing the access to iron by mycobacteria was suggested, which described the blockage of the synthesis pathway of the siderophores released by *M. tuberculosis* [121]. It is noteworthy that iron chelators are well-described competitive agents used against the pathogens’ iron acquisition [122], and they have also been indicated as a potential anti-inflammatory therapy for iron overload conditions, e.g., modulating inflammatory pathways and chelating iron [123]. Accordingly, the use of host-directed therapies with the purpose of protecting iron metabolism should focus on the players involved in iron trafficking and storage. In fact, the modulation of important key players in iron metabolism has already been described as beneficial during infectious and inflammatory conditions. Hepcidin modulation, for example, was proved to have a protective role during pulmonary infections, such as pneumonia [124], in autoimmune diseases, like lupus nephritis [125], or in infections caused by the siderophilic bacterium *Vibro vulnificus* through an induced hypoferremia mechanism [126].

**Figure 4:**
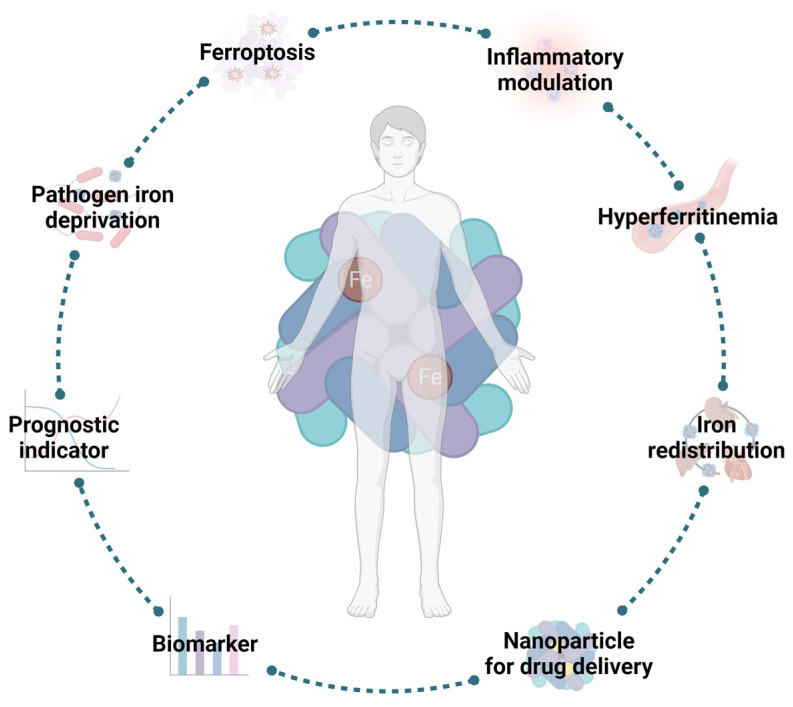
Ferritin in health and disease. Serum ferritin has a role in health and disease. Apart from its fully described role regarding iron storage, FT also responds to inflammatory stimuli, including infection. Its rising serum levels are associated with the development of deleterious conditions, such as ferroptosis or hyperferritinemia, which makes it an attractive target for the development of new host-directed therapies. However, these increased levels also give crucial information regarding the development of certain diseases or even their progression. Additionally, due to its nanocage structure, FT has also been used as a delivery system for drug therapies, such as vaccines. Image created with BioRender (www.biorender.com, accessed on 15 September 2023).

As previously mentioned, FT has a crucial role in health and disease (Figure 4) by modulating iron availability and signaling acute inflammatory states. Indeed, as an acute-phase protein, sFT expression is increased during inflammation. Additionally, FT also promotes the secretion of pro-inflammatory factors [53]. For that reason, FT, and, consequently, ferritinophagy, appear to be suitable targets for the development of new host-targeted therapies with the purpose of trying to reduce FT circulating levels by enhancing or diminishing the destruction of the protein. In fact, it is described that the activation of ferritinophagy may be used as an antitumoral mechanism in hepatic carcinoma, because the induction of ferroptosis will lead to the death of liver cancer cells [127]. The main protein involved in ferritinophagy is NCOA4 [128]. Thus, targeting ferritinophagy by impairing NCOA4 function appears to be a good strategy. Recent works have described NCOA4 as a novel therapeutical target to treat certain disorders, such as iron overload in a mouse model of hemochromatosis [129], pancreatic ductal adenocarcinoma [130], and infection by *M. tuberculosis* [131].

Nevertheless, we need to consider that FT has an important role in the modulation of iron metabolism, as it keeps iron within its core and releases it when in need. Therefore, it is possible that targeting FT for therapeutical purposes may endanger the iron balance, thereby dramatically raising serum iron levels and leading to iron overload disorders. Furthermore, increasing amounts of iron in circulation during infections will promote an easier way for pathogens to proliferate and survive, thus worsening disease recovery prospects. In contrast, the rising levels of FT provoked by the inflammatory condition will cause excessive iron accumulation within the protein, preventing its release into circulation and leading to the development of inflammatory anemia. Thus, targeting FT for therapy must consider not only the role of this protein in inflammation but also how the host’s iron metabolism will respond to alterations in the process of iron storage and distribution.

Nonetheless, FT appears to be a valuable protein to continue to be explored in the context of health and disease, as it has recently been described that FT may be used as a nanoparticle for therapy delivery, such as vaccine delivery to treat influenza and COVID-19 infections [132,133], as a nanocage loaded with antioxidative molecules to attenuate neurological degeneration [134], or as a novel detection system for the diagnosis of autoimmune diseases [135].

## 6. Conclusions

The first described role of ferritin as only an intracellular iron storage protein needs to be updated. Its role in iron homeostasis and as a potential biomarker in pathological conditions, including its potential in therapy, has gained relevance, although robust studies describing its direct clinical applicability as a biomarker are scarce. Nevertheless, recent links with disease progression and severity invigorate new discourse towards a better management of iron disturbances based on circulating ferritin levels. It is noteworthy that the understanding of how different proportions of H- and L-ferritin may affect tissue iron distribution appears to be a subject worthy of future research, because the redistribution of the different proteins’ subunits could have potential clinical implications in iron metabolism and iron-related disorders. With this review, we provide a comprehensive exploration of the role of ferritin in iron metabolism and give insights into its new functions.
